# Assessment of quality of life and its determinants in type-2 diabetes patients using the WHOQOL-BREF instrument in Bangladesh

**DOI:** 10.1186/s12902-022-01072-w

**Published:** 2022-06-18

**Authors:** Mohammod Feroz Amin, Bishwajit Bhowmik, Rozana Rouf, Monami Islam Khan, Syeda Anika Tasnim, Faria Afsana, Rushda Sharmin, Kazi Nazmul Hossain, Md. Abdullah Saeed Khan, Samiha Mashiat Amin, Md Shek Sady Khan, Md Faruque Pathan, Mohammad Jahid Hasan

**Affiliations:** 1 Department of Endocrinology, BIRDEM General Hospital, Dhaka, 1000 Bangladesh; 2 Diabetic Association of Bangladesh, Centre for Global Health Research, Dhaka, 1000 Bangladesh; 3 Square Hospital, Dhaka, Bangladesh; 4 Doctors in Life, Dhaka, 1000 Bangladesh; 5 Pi Research Consultancy Center, Dhaka, 1211 Bangladesh; 6 Shere Bangla Medical College, Barishal, Bangladesh; 7 Bangladesh University of Health Science, Mirpur, Dhaka, Bangladesh; 8 BIRDEM Academy, Dhaka, 1000 Bangladesh

**Keywords:** Quality of life, Type-2 diabetes mellitus, WHOQOL-BREF, Diabetic complications

## Abstract

**Background:**

Diabetes mellitus (DM) is rising at a rapid rate worldwide. As a chronic, incurable metabolic disease, diabetes affects a person’s life in all ways. Studies thus far have focused on the impact of diabetes on the physical and mental health of persons affected by the quality of life (QoL). This study aimed to explore the whole range of QoL deficits using the World Health Organization Quality of Life brief version (WHOQOL-BREF) in type-2 diabetic patients.

**Methods:**

This cross-sectional study was carried out among individuals aged at or above 15 years with type 2 diabetes (T2DM). Patients with prior mental health illness and unwillingness to give consent were excluded. A pretested structured questionnaire including the 26-item WHOQOL-BREF questionnaire was used for face-to-face interviews. Appropriate ethical measures were ensured. All statistical analyses were carried out using the statistical software STATA (Version 16.1). Graphs were created using R (Version 4.0.0).

**Results:**

A total of 500 T2 DM patients with a mean age of 55.8 ± 13.2 years (± SD) and a female proportion of 50.8% were included. Overall, 22.2% of participants rated their QoL as poor, and 25% were dissatisfied with their health (as assessed by questions 1 and 2 of the WHOQOL-BREF questionnaire). More than half (54% and 51.2%, respectively) had an average evaluation of their QoL and health. The QoL scores were below average, with mean scores (± SD) for the physical health, psychological, social relationship, and environmental domains of 37.2 ± 20.5, 44.2 ± 21.0, 39.6 ± 23.2, and 41.6 ± 19.5, respectively. Multiple regression analysis revealed that the patient’s level of education and monthly family income were significant positive modifiers and that complications (nephropathy, retinopathy, and peripheral artery disease) were significant negative determinants of the QoL score in different domains.

**Conclusion:**

This study found the overall quality of life among T2DM patients below average. Health authorities and clinicians should take these findings into account and incorporate necessary measures to ameliorate negative modifiers of the quality of life of sufferers.

**Supplementary Information:**

The online version contains supplementary material available at 10.1186/s12902-022-01072-w.

## Background

Type 2 diabetes mellitus (T2DM) is one of the four major non-communicable diseases (NCDs) rising at a rapid rate and causing a significant burden around the world [1, 2]. Global burden of disease estimates suggests that approximately 6.3% of the world’s population suffers from T2DM, with an expected rise in the coming years [3]. Like other countries around the globe, Bangladesh is also seeing an alarming increase in the prevalence of T2DM. A recent meta-analysis estimated a pooled prevalence of T2DM of approximately 7.8% in the general population in the country [4]. The high burden of T2DM threatens individuals’ QoL and existence.

T2DM is a chronic metabolic disease, the treatment of which is based primarily on lifestyle modification, exercise, and pharmacological agents [5]. The disease runs a chronic course and may lead to different micro- and macro-vascular complications [6]. In addition, patients are frequented by acute complications, including hypo- or hyperglycemic events, often requiring hospital admissions. Hypertension, obesity, and hyperlipidemia are the most frequent comorbidities in diabetic patients [7]. Additionally, T2DM has an evident relationship with many psychiatric illnesses, particularly depressive disorders [8]. Moreover, long-term treatment adherence means a significant cost burden for patients and their families [9]. Hence, a coordinated effort of health care personnel, patients, and their families are required to achieve desirable control of the disease. Consequently, T2DM affects a person not only physically but also psychologically, socially, and economically, exerting a negative impact on their QoL.

The impact of diabetes on patients’ QoL has been studied extensively [10, 11], and T2DM was proven to reduce patients’ health-related quality of life (HR-QoL) [11]. The majority of these studies focused on the physical and mental aspects of QoL patients. Previous studies in Bangladesh used the EuroQol-5 Dimensions Questionnaire (EQ-5D) [12], a generic measure to assess HR-QoL [13, 14]. However, the short form of the World Health Organization Quality of Life assessment instrument (WHOQOL-BREF) probes QoL in four domains, namely, physical, psychological, social, and environmental domains [15]. As diabetes affects a person’s life in every direction, this study aimed to explore these four domains of QoL in people with T2DM using WHOQOL-BREF and determine its associated factors.

## Methods

### Study design, participants, and place

This cross-sectional study was carried out in the outpatient department of Bangladesh Institute of Research and Rehabilitation in Diabetes, Endocrine and Metabolic Disorders (BIRDEM), a specialized center for diabetes and endocrine disease management and research in Bangladesh. This study was conducted over a period of 6 months, from January to June 2019. Young adults and adults (≥ 15 years) diagnosed with T2DM by oral glucose tolerance test (OGTT) were approached for inclusion. Patients with prior mental health illness and those unwilling to participate were excluded. We used the following formula for sample size calculation. 

$\mathrm n=\frac{{Z^2}_{1-{\displaystyle\frac\alpha2}}pq}{d^2}$where *n* = sample size, ${Z}_{1-\frac{\alpha }{2}}$ =Standard deviate at desired level of significance, *p* = sample proportion, q = 1-p, and d = margin of error. From the study by Barua et al. [14], using the prevalence of an average EQ-5D-5L index score of 53.4% (*p* = 0.534), 95% level of significance (Z_1/α_ = 1.96), and 5% margin of error (d = 0.05), we calculated the sample size to be 382.38 (~ 383). However, a total of 500 patients with T2DM were conveniently included during the study period. Which lowered the margin of error to 4.37% when level of significance remains 95% or upgraded the level of significance to 98.75% when margin of error remains 5%. There were no missing data. 

### Study instrument

The study instrument used to collect data from patients was a pretested structured questionnaire (Supplementary file 1) produced after extensive review of previous literature and discussion with experts. The questionnaire consisted of sociodemographic information, diabetes-related information, comorbidities, complications, investigations, and QoL assessment. The 26-item World Health Organization (WHO)-endorsed QoL questionnaire (brief version), known as WHOQOL-BREF [15], was adapted for the QoL part. The validated Bangla version of the WHOQOL-BREF [16] was used with permission from the original authors.

### Sociodemographic profile

The sociodemographic section of the questionnaire obtained information on patients’ age, sex, education, employment, and monthly family income.

### Diabetes-related information, comorbidity profile, and complications

The second section comprised questions related to patients’ diabetes, asking about the duration of diabetes, name, and duration of anti-diabetic agents used. Patients’ body mass index (BMI) was calculated from their height and weight at the time of the interview, and they were asked for a history of hypertension (HTN). Documented complications were recorded in this section. The complications queried were nephropathy, retinopathy, neuropathy, stroke, coronary artery disease (CAD), peripheral artery disease (PAD), and diabetic foot.

### Biochemical profile

Glycemic profile (fasting blood sugar [FBS], blood sugar 2 h after breakfast [2hABF], HbA1c), lipid profile (total cholesterol [TC], low-density lipoprotein [LDL], high-density lipoprotein [HDL], and triglyceride [TG]), and renal function (serum creatinine, urinary albumin-creatinine ratio [ACR]) were investigated and recorded. FBS and 2HABF were assessed using Hexokinase method by Beckman Coulter Au-680 auto analyzer, serum lipid profile were tested by ARCHITECT c4000 Clinical Chemistry Analyzer, serum creatinine was measured using analyzer name and ACR was determined by BN ProSpec,SIMENS.

### WHOQOL-BREF

The WHOQOL-BREF to measure the QoL of T2DM patients used in this study is a validated short version of the WHOQOL-100 quality of life assessment instrument [17]. It is a generic instrument that can be applied cross-culturally. The questionnaire assesses QoL in four domains, namely, the physical health, psychological, social relationships, and environment domains, and thus covers the whole range of QoL deficits. It has a simple response format and allows fine-grained discrimination of QoL across individuals. Considering the points to be noted during a cross-sectional assessment of QoL [17], we found that WHOQOL-BREF best fits our purpose.

### Study procedure

Trained research assistants conducted a face-to-face interview of the patients outdoors after giving consent for inclusion. Before interviews, they explained the nature and purpose of the study to the participant. Misunderstood items were simply repeated, and respondents were encouraged to interpret the questions in their way. The WHOQOL-BREF part of the questionnaire was scored in accordance with the manual [15]. The WHOQOL-BREF showed excellent internal consistency among our respondents (Cronbach’s alpha coefficient = 0.97).

### Statistical analysis

Data from all participants were entered into a Microsoft Excel file first and then was imported to statistical software for analysis. There were no missing data. We used descriptive and inferential methods to determine the impact of diabetes on QoL. Normality assumptions were checked, and analysis of variance (ANOVA), independent sample t-test, and Mann–Whitney U test were used to compare continuous variables among different groups. Continuous variables were expressed as the mean ± standard deviation and median (interquartile range [IQR]) wherever applicable. Categorical variables were described by frequencies (percent), and chi-square tests were used to identify associations between groups. All tests were two-tailed, and p values less than or equal to 0.05 were considered statistically significant. QoL scores were transformed to a 100-point scale using methods detaliled in WHOQOL-BREF manual [15]. The internal consistency of QoL scores, measured by the WHOQOL-BREF instrument, was assessed using Cronbach’s alpha. Multiple linear regression analysis was used to determine factors independently associated with different domains of QoL. Prior to modelling for each individual domain, we checked multicollinearity using variance inflation factor (VIF) (which was < 10 for all models) and condition index (which was < 15 for all independent variables), and auto-correlation of residuals using Durbin Watson statistic (which ranged from 1.2 to 2.0 for all models). The final models were built when all the assumptions were met. We used Statistical software STATA (Version 16.1) and R (Version 4.0.0) for statistical analysis and graphs.

### Ethical consideration

Ethical approval for this study was obtained from the ethical review committee (ERC) of BIRDEM. All procedures were conducted following the ethical standards of the current Declaration of Helsinki. Informed written consent was taken before participation of the study.

## Results

A total of 500 patients with T2DM participated in the study. The average age of the participants was 55.8 ± 13.2 years, with an IQR of 46 – 65 years. Patients aged 51–60 years constituted the highest percentage (27.4%) among the different age groups. Slightly more than half of the patients were female (50.8%). Most patients had education up to a higher secondary level (57.5%), were part-time employed, and had a monthly income between 5000 and 25,000 BDT (59.2%). The mean duration of diabetes was 11.3 ± 7.9 years (SD), and an average of 1.8 ± 3.3 years (SD) passed without medication. Rapid-acting insulin was the most widely used hypoglycemic agent (36.8%) among patients. HTN was present in 70.4% of patients, and 76% of patients were overweight/obese. Of all, 47% of patients had at least one complication, nephropathy (29%) was the most common microvascular complication, and coronary artery disease (13%) was the most common macrovascular complication among patients (Table 1).

**Table 1:** Sociodemographic profile, duration of diabetes, medication history, comorbidities and complications of patients

Variables	n (%)
**Age (years)**	
Mean ± SD	55.85 ± 13.24
Median (IQR)	55 (46 – 65)
Age group	
16 – 30	12 (2.4)
31 – 40	54 (10.8)
41 – 50	131 (26.2)
51 – 60	137 (27.4)
61 – 70	112 (22.4)
71 – 80	39 (7.8)
> 80	15 (3.0)
**Sex**	
Female	254 (50.8)
Male	246 (49.2)
**Education**^a^	
Uneducated	110 (22.04)
Up to higher secondary	282 (57.51)
Graduate	80 (16.03)
Postgraduate	27 (5.41)
**Employment**^a^	
No job	131 (26.25)
Part time	212 (42.48)
Full time	111 (22.24)
Retired	45 (9.02)
**Monthly family Income (BDT)**	
< 5000	84 (16.8)
5000—25,000	296 (59.2)
> 25,000	120 (24.0)
**Duration of diabetes (years)**	
Mean ± SD	11.27 ± 7.86
Median (IQR)	10 (5 – 15)
Categories	
≤ 10	268 (53.6)
11 – 20	181 (36.2)
> 20	51 (10.2)
**Years passed without medication**	
Mean ± SD	1.79 ± (3.26)
Median (IQR)	0 (0 – 3)
Categories	
None to < 1	311 (62.2)
1—< 3	63 (12.6)
3—< 5	45 (9.0)
≥ 5	81 (16.2)
**Hypoglycemic agents used**^b^	
Metformin	47 (9.4)
Sulfonylureas	21 (4.2)
Alpha glucosidase	2 (0.4)
DPP IV inhibitors	17 (3.4)
Rapid-acting Insulin	184 (36.8)
Intermediate-acting insulin	140 (28.0)
Long-acting insulin	7 (1.4)
Mixed insulin	109 (21.8)
**Comorbidities**^b^	
Hypertension	352 (70.4)
Overweight/obesity	379 (76.0)
**Complications**^b^	
* Microvascular complications*	
Nephropathy	145 (29.0)
Neuropathy	108 (21.6)
Retinopathy	51 (10.2)
* Macrovascular complications*	
Coronary artery disease	65 (13.0)
Stroke	26 (5.2)
Peripheral artery disease	21 (4.2)
Diabetic foot	53 (10.6)
* Acute complication*	
History of hypoglycemia	290 (58.0)
* Number of complications*	
None	265 (53.0)
One	115 (23.0)
Two	56 (11.2)
Three or more	64 (12.8)

^a^ After excluding missing values

^b^ Multiple response considered

Table 2 describes the investigation profile of patients. The average FBS, 2hABF, and HbA1c were 10.3 ± 4.0 mmol/l (SD), 14.8 ± 13.3 mmol/l (SD) and 10.1 ± 2.7% (SD), respectively. Of all, 77.6% had FBS > 7 mmol/L, 92.2% had 2hABF > 8 mmol/L and 93.4% had HbA1c > 6.5%. Moreover, 54.6% of patients had an ACR between 30 and 300 mcg/mg, and 23.4% had a ratio > 300. Serum TC was ≥ 200 in 36% of patients, LDL was ≥ 100 in only 0.2%, HDL was > 40 (male)/ > 50 (female) in 91.8% and TG was ≥ 150 in 75.6% patients (all units in mg/dl).

**Table 2:** Biochemical profile of patients

Variable	Properties	n (%)
Fasting blood glucose (mmol/l)	Mean ± SD	10.25 ± 3.97
	Median (IQR)	10 (7.3 – 12.2)
	≤ 7.00	112 (22.4)
	> 7.00	388 (77.6)
Blood sugar 2 h after breakfast (mmol/l)	Mean ± SD	14.82 ± 13.25
	Median (IQR)	13 (10.3 – 16.8)
	≤ 8.00	39 (7.8)
	> 8.00	461 (92.2)
HbA1c (%)	Mean ± SD	10.07 ± 2.73
	Median (IQR)	9.8 (8 – 1)
	≤ 6.5	33 (6.6)
	> 6.5	467 (93.4)
Serum creatinine (mg/dl)	Mean ± SD	1.31 ± (0.99)
	Median (IQR)	1.04 (0.9 – 1.3)
	< 1.5	420 (84.0)
	≥ 1.5	80 (16.0)
Urinary albumin-creatinine ratio (mcg/mg)	Mean ± SD	388.74 ± (7.00 – 8457)
	Median (IQR)	121 (45 – 258)
	< 30	110 (22.0)
	30 – 300	273 (54.6)
	> 300	117 (23.4)
Serum total cholesterol (mg/dl)	Mean ± SD	185.30 ± 68.46
	Median (IQR)	173 (138 – 228)
	< 200	320 (64.0)
	≥ 200	180 (36.0)
Serum low density lipoprotein (mg/dl)	Mean ± SD	42.76 ± 33.12
	Median (IQR)	21 (13 – 77.5)
	< 100	499 (99.8)
	≥ 100	1 (0.2)
Serum high density lipoprotein (mg/dl)	Mean ± SD	30.9 ± 10.10
	Median (IQR)	30 (24 – 36)
	> 40 (for male) or > 50 (for female)	41 (8.2)
	≤ 40 (for male) or ≤ 50 (for female)	459 (91.8)
Serum triglyceride (mg/dl)	Mean ± SD	312.87 ± 263.38
	Median (IQR)	214 (151 – 329)
	< 150	122 (24.4)
	≥ 150	378 (75.6)

**Fig. 1:**
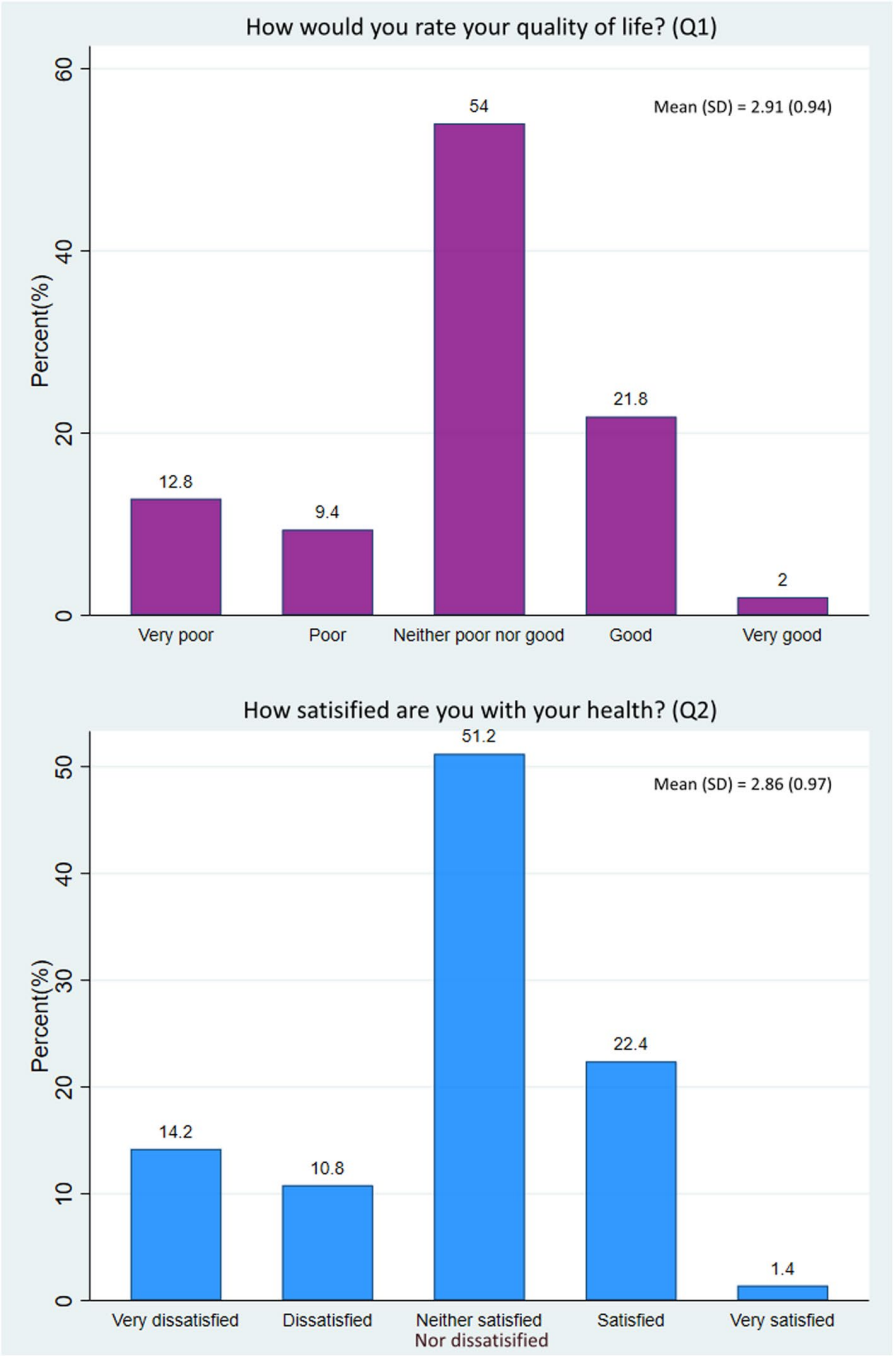
Patients’ overall perception and satisfaction about their quality of life and health (Question no 1 and 2 of WHOQOL-BREF scale) [SD: Standard Deviation]

Question no. 1 (How would you rate your quality of life?) and 2 (How satisfied are you with your health?) of the WHO-BREF questionnaire assesses individuals’ overall perception about their QoL and satisfaction level regarding their health, respectively. Among our patients, 22.2% thought that they had an overall poor QoL. One-quarter of patients had general dissatisfaction about their health (Fig. 1). The average score was 2.9 (SD 0.94) for the first question and 2.9 (SD 0.97) for the second question.

**Fig. 2:**
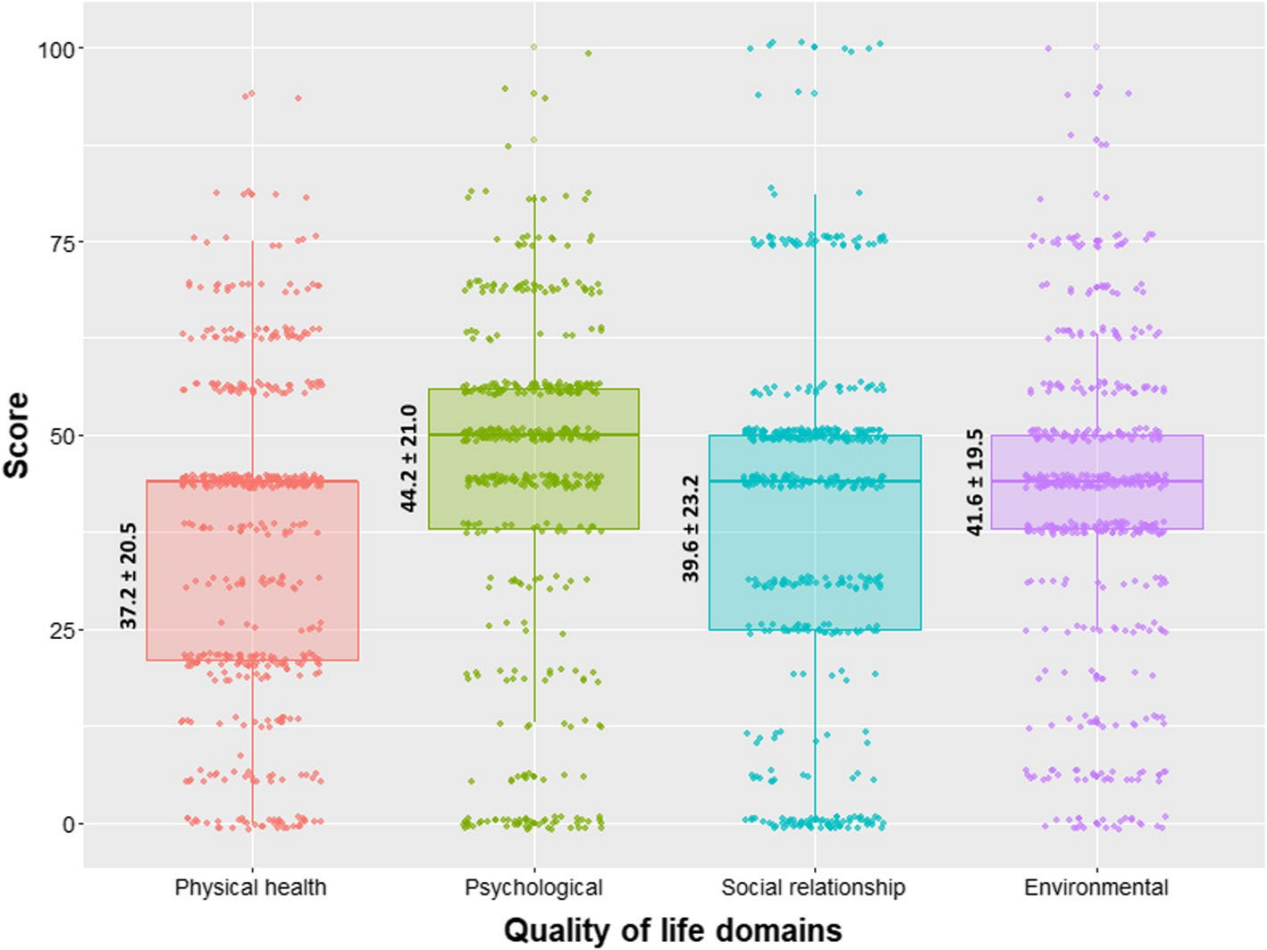
Jitter boxplot showing the quality of life domain scores of participants. [Mean ± standard deviation is shown on the left side of each boxplot. The median domain score with interquartile range (IQR) is as follows: physical domain score: 44 (21 – 44); psychological domain score: 50 (38 – 56); social relationship domain score: 44 (25 – 50); environmental domain: 44 (38 – 50)]

The mean score with SD and median scores with IQR of the four domains of QoL are shown in Fig. 2. The median score was lower than 50 for the physical health, psychological, social relationship, and environmental domain scores. However, it was higher for the psychological domain than for the other domains. However, the distribution of scores (as measured by IQR) was higher for social relationship scores than for other domains.

The four QoL domain scores (at a scale of 100) were compared across categories of different patient characteristics and are presented in Table 3. QoL scores were similar among age categories across all domains except the psychological domain. The score was significantly lower (*p* = 0.027) for patients aged > 80 years compared to most other age groups for the latter domain. A similar distribution of scores was noted between males and females. The average psychological domain score was significantly lower in females than in males (*p* = 0.044). All domain scores were low in uneducated patients and showed a statistically significant graded increase with education levels (*p* < 0.05 for all domains). Retired patients had significantly lower QoL scores in all domains than employed and even unemployed individuals (*p* < 0.001 for all domains). A higher monthly family income was associated with a higher QoL score (*p* < 0.001 for all domains). Patients who had T2DM for > 20 years had a significantly low QoL score in all four domains (*p* < 0.05). HTN was associated with a significantly low physical domain score (*p* = 0.02). On the other hand, being overweight or obese was associated with a low social relationship and environmental domain score (*p* = 0.037 and *p* = 0.0.38, respectively). A high serum cholesterol level (≥ 200 mg/dl) was associated with significantly lower physical health, psychological and social relationship domain scores than a low value (*p* = 0.029, *p* = 0.007, and *p* = 0.016, respectively). All microvascular (nephropathy, retinopathy, and neuropathy) and macrovascular complications (CAD, stroke, and PVD) were associated with significantly low QoL scores in all domains (*p* < 0.05 for all). The score declined significantly with the combination of complications among patients (*p* < 0.001).

**Table 3:** Four quality of life domain scores across different characteristics of participants

Variables	Physical health domain	Psychological domain	Social relationship domain	Environmental domain
	**Mean ± SD**	**Mean ± SD**	**Mean ± SD**	**Mean ± SD**
**Age groups**				
16 – 30	35.91 ± 23.51	41.67 ± 24.08	39.00 ± 21.38	36.08 ± 23.74
31 – 40	40.70 ± 21.99	48.22 ± 22.08	42.44 ± 24.45	43.79 ± 21.33
41 – 50	40.94 ± 21.47	46.34 ± 20.18	41.11 ± 24.23	44.10 ± 19.55
51 – 60	35.36 ± 20.55	42.92 ± 22.15	39.49 ± 23.53	42.39 ± 19.91
61 – 70	34.21 ± 19.50	42.79 ± 20.51	38.72 ± 23.333	38.72 ± 19.54
71 – 80	37.97 ± 13.87	46.92 ± 11.91	38.72 ± 23.33	40.77 ± 10.33
> 80	31.40 ± 21.19	28.00 ± 25.48^bcd^	40.77 ± 13.34	33.80 ± 21.58
*p* value	0.096	**0.027**	0.078	0.178
**Sex**				
Female	36.90 ± 20.75	42.33 ± 21.80	38.35 ± 24.02	41.32 ± 20.63
Male	37.59 ± 20.25	46.11 ± 19.92	40.88 ± 22.20	41.95 ± 18.35
*p* value	0.708	**0.044**	0.223	0.719
**Education**				
Uneducated	30.61 ± 19.50	37.02 ± 21.85	33.04 ± 23.92	36.17 ± 21.88
Up to HSC	37.70 ± 19.47^a^	45.26 ± 19.09^a^	41.25 ± 22.87^a^	41.74 ± 17.68
Graduate	42.15 ± 22.36^a^	48.75 ± 21.24^a^	41.65 ± 22.87	45.65 ± 19.07^a^
Post-graduate	46.26 ± 21.04^a^	50.48 ± 26.70^a^	43.66 ± 28.70	51.85 ± 22.62^a^
*p* value	** < 0.001**	** < 0.001**	**0.008**	** < 0.001**
**Employment**				
No job	39.24 ± 18.34	45.59 ± 16.31	40.29 ± 18.68	40.88 ± 16.20
Part time	37.92 ± 18.70	45.77 ± 19.25	41.01 ± 20.44	43.00 ± 16.90
Full time	39.82 ± 23.40	45.83 ± 24.19	42.41 ± 27.82	45.03 ± 23.29
Retired	21.73 ± 21.03^abc^	28.51 ± 26.07	23.87 ± 28.75^abc^	29.07 ± 24.93^abc^
*p* value	** < 0.001**	** < 0.001**	** < 0.001**	** < 0.001**
**Monthly family income (BDT)**				
< 5000	26.84 ± 22.85	30.65 ± 24.66	27.12 ± 26.81	31.26 ± 24.83
5000 – 25,000	38.08 ± 20.32^a^	45.23 ± 20.17^a^	40.80 ± 22.97^a^	42.71 ± 18.80^a^
> 25,000	42.43 ± 16.33^a^	51.11 ± 15.14^ab^	45.37 ± 17.07^a^	46.24 ± 13.86^a^
*p* value	** < 0.001**	** < 0.001**	** < 0.001**	** < 0.001**
**Duration of diabetes mellitus (years)**				
≤ 10	41.19 ± 18.49	46.68 ± 17.85	42.20 ± 20.05	43.85 ± 17.14
11 – 20	33.77 ± 21.20^a^	42.49 ± 23.33	37.75 ± 25.67	40.12 ± 21.06
> 20	28.76 ± 23.11^a^	37.17 ± 25.10^a^	32.47 ± 27.03^a^	35.29 ± 23.73^a^
*p* value	** < 0.001**	**0.005**	**0.009**	**0.007**
**Years passed without medication (years)**				
No to < 1	36.86 ± 19.65	44.23 ± 20.01	39.79 ± 21.68	40.81 ± 18.49
1 to < 3	38.54 ± 21.19	46.67 ± 22.19	38.41 ± 24.63	42.63 ± 19.17
3 to < 5	37.98 ± 21.42	43.6 ± 22.61	39.15 ± 26.74	45.47 ± 23.13
≥ 5	37.27 ± 22.78	42.45 ± 22.73	40.00 ± 25.64	41.89 ± 21.52
*p* value	0.937	0.690	0.973	0.482
**Hypertension**				
Present	35.87 ± 21.49	42.99 ± 20.56	38.47 ± 22.08	40.76 ± 18.73
Absent	40.51 ± 19.92	47.05 ± 21.67	42.27 ± 25.41	43.70 ± 21.23
*p* value	**0.020**	0.050	0.094	0.126
**Overweight/obesity**				
Present	38.11 ± 19.99	45.24 ± 20.82	40.88 ± 22.54	42.72 ± 18.77
Absent	34.82 ± 21.68	41.26 ± 20.89	35.85 ± 24.51	38.49 ± 21.33
*p* value	0.125	0.069	**0.037**	**0.038**
**HbA1c (%)**				
> 6.5	37.75 ± 20.62	44.39 ± 20.93	39.76 ± 23.15	41.73 ± 19.67
≤ 6.5	30.09 ± 17.17	41.39 ± 21.59	37.27 ± 23.36	40.15 ± 17.68
*p* value	**0.038**	0.428	0.551	0.653
**Total cholesterol (mg/dl)**				
< 200	38.73 ± 36.65	46.08 ± 19.18	41.47 ± 20.97	42.34 ± 17.53
≥ 200	34.58 ± 22.87	40.85 ± 23.48	36.27 ± 26.33	40.38 ± 22.64
*p* value	**0.029**	**0.007**	**0.016**	0.282
**Serum high density lipoprotein (mg/dl)**				
≥ 40 (for male) or ≥ 50 (for female)	32.80 ± 19.98	43.02 ± 22.81	36.75 ± 21.74	37.68 ± 17.37
< 40 (for male) or < 50 (for female)	37.64 ± 20.50	44.80 ± 20.81	39.84 ± 23.28	41.98 ± 19.68
*p* value	0.148	0.709	0.412	0.177
**Serum triglyceride (mg/dl)**				
< 150 mg/dl	40.09 ± 19.58	48.77 ± 18.92	42.94 ± 20.57	44.21 ± 17.71
≥ 150 mg/dl	36.33 ± 20.71	42.71 ± 21.39	38.52 ± 23.85	40.79 ± 20.03
*p* value	0.082	**0.005**	0.066	0.093
**Nephropathy**				
Present	28.93 ± 20.94	36.15 ± 23.95	31.18 ± 24.36	34.12 ± 21.09
Absent	40.63 ± 19.31	47.47 ± 18.67	43.03 ± 21.76	44.70 ± 18.00
*p* value	** < 0.001**	** < 0.001**	** < 0.001**	** < 0.001**
**Neuropathy**				
Present	29.40 ± 22.49	46.26 ± 19.30	33.88 ± 28.90	36.37 ± 23.39
Absent	39.40 ± 19.38	36.69 ± 24.80	41.17 ± 21.08	43.08 ± 18.09
*p* value	** < 0.001**	** < 0.001**	0.029	**0.002**
**Retinopathy**				
Present	19.27 ± 22.67	23.70 ± 25.27	20.06 ± 28.86	26.12 ± 25.65
Absent	39.28 ± 19.21	46.52 ± 19.10	41.81 ± 21.35	43.39 ± 17.91
*p* value	< 0.001	< 0.001	< 0.001	< 0.001
**Coronary artery disease**				
Present	23.81 ± 20.12	28.18 ± 24.55	25.43 ± 25.28	31.12 ± 23.94
Absent	39.25 ± 19.79	46.59 ± 19.29	41.71 ± 22.08	43.20 ± 18.30
*p* value	** < 0.001**	** < 0.001**	** < 0.001**	** < 0.001**
**Stroke**				
Present	22.92 ± 16.68	27.96 ± 22.41	24.5 ± 24.13	29.88 ± 20.91
Absent	38.02 ± 20.39	45.08 ± 20.53	40.42 ± 22.84	42.28 ± 19.26
*p* value	** < 0.001**	** < 0.001**	**0.001**	**0.002**
**Peripheral artery disease**				
Present	17.66 ± 22.26	23.95 ± 25.63	13.95 ± 24.74	20.19 ± 19.09
Absent	28.09 ± 19.99	45.08 ± 20.31	40.72 ± 22.45	42.57 ± 19.02
*p* value	** < 0.001**	** < 0.001**	** < 0.001**	** < 0.001**
**Diabetic foot**				
Present	28.81 ± 21.90	39.15 ± 24.40	31.69 ± 27.88	33.00 ± 21.13
Absent	38.29 ± 20.10	44.79 ± 20.46	40.53 ± 22.38	42.66 ± 19.10
*p* value	**0.004**	0.132	0.052	**0.007**
**History of hypoglycemia**				
Present	37.20 ± 19.45	44.33 ± 20.27	39.46 ± 21.59	40.62 ± 18.51
Absent	37.30 ± 21.88	44.00 ± 21.92	39.78 ± 25.21	43.02 ± 20.82
*p* value	0.974	0.860	0.431	**0.027**
**Number of complications**				
None	42.08 ± 18.41	48.70 ± 17.52	43.30 ± 19.78	45.24 ± 16.58
One	40.03 ± 18.27	42.43 ± 20.89^a^	39.73 ± 24.08^a^	45.17 ± 17.93
Two	28.34 ± 21.48^ab^	35.64 ± 26.34^a^	30.22 ± 28.35^a^	33.73 ± 21.14^ab^
Three or more	19.97 ± 20.22^ab^	16.42 ± 21.54^abc^	11.71 ± 21.99^abc^	27.27 ± 23.56^ab^
*p* value	** < 0.001**	** < 0.001**	** < 0.001**	** < 0.001**

The *p* value was determined by independent samples t test, Mann–Whitney U test, and one-way analysis of variance where appropriate. Post hoc pairwise comparisons were done using Tukey. For pairwise comparisons, the *p* value was significant at the < 0.05 level compared to the ^a^first category, ^b^second category, ^c^third category and ^d^sixth category

Significant values were shown in bold face

After adjustment for sociodemographic characteristics, comorbidity and complication-related variables in multiple linear regression analysis, different sets of factors were found to be significantly associated with the four separate domains of the WHO-BREF QoL scale (Table 4). In the physical domain, patients’ level of education and the presence of diabetic nephropathy and retinopathy were significant predictors. Compared to patients with no education, the physical domain score was 6.368 higher (*p* = 0.006) for patients educated up to Higher Secondary Certificate (HSC), 10.893 higher (*p* = 0.001) for graduates, and 15.687 higher (*p* = 0.001) for postgraduates. The presence of nephropathy and retinopathy was associated with 4.764-point (*p* = 0.028) and 8.859-point (*p* = 0.011) decreases in the physical domain score, respectively. The psychological domain was 3.113 points (*p* = 0.029) higher in patients with graduation than those without education. A monthly family income between 5000 and 25,000 BDT and > 25,000 BDT was associated with 8.121 points (*p* = 0.002), and 10.159 points (*p* = 0.002) increases in the psychological domain score compared to < 5000 BDT. The presence of retinopathy, CAD, and PAD was associated with significantly low psychological domain scores (β = -12.003, *p* = 0.001; β = -7.523, *p* = 0.013; and β = -12.231, *p* = 0.022, respectively). The social relationship domain score was significantly higher with higher monthly family income (β = 7.621, *p* = 0.012 and β = 8.925, *p* = 0.016 for income groups 5000 – 25,000 and > 25,000, respectively). The presence of retinopathy and PAD was associated with a significant decrease in the social relationship domain score (β = -10.962, *p* = 0.006 and β = -18.14, *p* = 0.003). For the environmental domain, an education level up to graduation (β = 7.963, *p* = 0.008) and postgraduation (β = 14.822, *p* = 0.001), a monthly income between 5000 and 25,000 (β = 6.605, *p* = 0.009), and an HDL < 40 (male)/ < 50 (female) (β = 7.055, *p* = 0.023) were significant predictors of a higher score. The presence of nephropathy, retinopathy, and PAD were significant negative predictors of the environmental domain score (β = -5.279, *p* = 0.015; β = -8.618, *p* = 0.01 and β = -13.867, *p* = 0.007, respectively).

**Table 4:** Multiple linear regression showing factors associated with four domains of quality of life among patients

	**Quality of life domains**							
	**Physical health**		**Psychological**		**Social relationship**		**Environmental**	
	**Adjusted β (95%CI)**	***p* value**	**Adjusted β (95%CI)**	***p* value**	**Adjusted β (95%CI)**	***p* value**	**Adjusted β (95%CI)**	
**Age**	-0.006	0.935	-0.079	0.309	-.100	0.263	-0.032	0.673
**Sex**								
** Female**	Ref		Ref		Ref		Ref	
** Male**	-1.371	0.458	2.302	0.212	2.520	0.235	0.077	0.966
**Education**								
** No education**	Ref		Ref		Ref		Ref	
** Up to HSC**	6.368	**0.006**	4.310	0.064	5.259	**0.049**	4.051	0.071
** Graduate**	10.893	**0.001**	3.113	**0.029**	4.730	0.187	7.963	**0.008**
** Postgraduate**	15.687	**0.001**	8.651	0.055	6.753	0.193	14.822	**0.001**
**Employment**								
** No employment/Retired**	Ref		Ref		Ref		Ref	
** Part-time/full-time job**	-.506	0.798	0.273	0.890	.871	0.701	1.681	0.377
**Monthly family income (BDT)**								
** < 5000**	Ref		Ref		Ref		Ref	
** 5000 – 25,000**	5.154	0.050	8.121	**0.002**	7.621	**0.012**	6.605	**0.009**
** > 25,000**	4.307	0.182	10.159	**0.002**	8.925	**0.016**	5.269	0.090
**Duration of diabetes (years)**								
** ≤ 10**	Ref		Ref		Ref		Ref	
** 11–20**	-3.664	0.053	0.585	0.757	0.238	0.913	-0.063	0.973
** > 20**	-3.851	0.224	0.644	0.838	0.458	0.900	0.326	0.915
**Hypertension**								
** Absent**	Ref		Ref		Ref		Ref	
** Present**	-1.516	0.459	-1.657	0.418	-1.441	0.540	-0.114	0.954
**Overweight/obesity**								
** Absent**	Ref		Ref		Ref		Ref	
** Present**	1.575	0.445	2.271	0.270	3.478	0.142	2.197	0.268
**Total cholesterol (mg/dl)**								
** < 200**	Ref		Ref		Ref		Ref	
** ≥ 200**	-1.748	0.445	-2.857	0.131	-3.040	0.162	0.196	0.914
**High density lipoprotein (mg/dl)**								
** ≥ 40 (male)/ ≥ 50 (female)**	Ref		Ref		Ref		Ref	
** < 40 (male)/ < 50 (female)**	5.953	0.064	3.825	0.234	5.363	0.146	7.055	**0.023**
**Triglyceride (mg/dl)**								
** < 150**	Ref		Ref		Ref		Ref	
** ≥ 150**	-2.187	0.278	-3.824	0.058	-2.144	0.354	-2.250	0.246
**Nephropathy**								
** Absent**	Ref		Ref		Ref		Ref	
** Present**	-4.764	**0.034**	-2.909	0.194	-4.675	0.069	-5.279	**0.015**
**Neuropathy**								
** Absent**	Ref		Ref		Ref		Ref	
** Present**	-0.838	0.727	0.219	0.927	3.350	0.225	1.425	0.538
**Retinopathy**								
** Absent**	Ref		Ref		Ref		Ref	
** Present**	-8.859	**0.011**	-12.003	**0.001**	-10.962	**0.006**	-8.618	**0.010**
**Coronary artery disease**								
** Absent**	Ref		Ref		Ref		Ref	
** Present**	-5.134	0.089	-7.523	**0.013**	-5.129	0.138	-3.067	0.291
**Stroke**								
** Absent**	Ref		Ref		Ref		Ref	
** Present**	-2.159	0.600	-2.208	0.591	-2.238	0.636	-1.469	0.711
**Peripheral artery disease**								
** Absent**	Ref		Ref		Ref		Ref	
** Present**	-8.763	0.102	-12.231	**0.022**	-18.14	**0.003**	-13.867	**0.007**
**Diabetic foot**								
** Absent**	Ref		Ref		Ref		Ref	
** Present**	-3.318	0.336	1.334	0.698	-1.399	0.724	-3.524	0.289

## Discussion

Our study revealed that the overall QoL of T2DM patients in the physical health, psychological, social relationship, and environmental domains was at or below the middle (i.e., 50) of the possible score range (0 – 100 for WHOQOL-BREF at a scale of 100 [15]). In addition, individuals’ perception of their QoL and health was predominantly average (i.e., 50). Nearly one-quarter rated their QoL as poor, and one-quarter were unsatisfied with their physical health. Patient education and monthly family income were significant positive modifiers of QoL. At the same time, the presence of complications was an important negative determinant of QoL scores.

Mishra et al. [18] used the WHOQOL-BREF scale to assess QoL among T2DM patients in Nepal. They reported mean QoL scores (± SD) of 50.7 ± 11.8, 53.3 ± 10.3, 57.3 ± 8.9, and 54.7 ± 7.7 in the physical health, psychological, social relationship, and environmental domains, respectively, among T2DM patients. However, the scores were 37.2 ± 20.5, 44.2 ± 21.0, 39.6 ± 23, and 41.6 ± 19.5 in our study, pointing to a low QoL score in this group of patients. Similarly, in West Java, Puspasari and Farera [19] and Abbasi-Ghahramanloo et al. [20] in Iran observed that most T2DM patients had poor QoL in three doamins – physical, psychological, and environmental. These findings imply that T2DM not only affects persons’ physical health, but also their mental well-being, social relationships, and environmental factors like financial resources, accessibility of care, recreation/leisure activity and home environment, among others. One study conducted in India [21] suggests that all domains of QoL scores were significantly higher in patients with controlled DM compared to patients with uncontrolled DM. In Bangladesh no previous study used WHOQOL-BREF to assess the QoL of T2DM patients. However, a study developed by Safita et al. [22], although using the EQ-5D-3L instrument, found that the negative impact of T2DM in health-related QoL of patients might be significantly high, when compared to non-diabetic individuals. The EQ-5D instruments [23], developed by EuroQoL Research Foundation, assess QoL in five dimensions, namely, mobility, self-care, usual activities, pain/discomfort, and anxiety/depression. It has two versions for adults: EQ-5D-3L and EQ-5D-5L, which allows an individual to choose from three and five levels of subjective assessment points for each dimension. As management of T2DM and associated complications requires a long-term commitment to discipline, dietary control, and drugs which requires support from family and friends, diagnosing with T2DM creates a need for comprehensive support from all aspects. Feng and Astell-Burt [24] reported that a diagnosis of T2DM was associated with a reduction in time spent with family and friends, contacts by telephone, and social or religious gatherings. Which entails a further reduction in QoL in a positive feedback cycle.

Various factors work in concert to determine the QoL of diabetic patients. However, as Trikkalinou et al. [11] observed, these factors vary based on the study subject, design, and methods used. We did not find any significant variations in QoL scores across age groups in any domains except the psychological domain, where the eldest group of patients had a significantly lower score. However, after adjustment for other factors, we did not find any association of age with QoL scores in any domain. This is unlike Gebremedhin, Workicho and Angaw [25], who noted a significant association of age with all domains of QoL They found an age-related decline in HRQoL among T2DM patients. We did not notice any association of QoL with sex of the patients. On the contrary Manjunath et al. [26] found that being female was associated with poor quality of life. One possible explanation of these differences is the combined adjustment of socioeconomic variables and complications in our regression model, which was considered in a few preceding studies. It is well established that the number of micro- and macrovascular complications increases with the duration of diabetes [27]. Hence, the impact of patients’ age and duration of diabetes is expected to attenuate when complications are taken into consideration with respect to QoL measures. We also noted that after adjustment for other factors in our study, the impact of the duration of diabetes on QoL faded, which is similar to that of Manjunath et al. [26],. However, longer duration of diabetes was found to be significantly associated with poorer QoL among elderly diabetics [28].

Similar to Esin et al. [29], we found a significant positive association of QoL with monthly family income. Patients’ psychological, social relationship, and environmental domain scores were significantly higher in the high-income groups. Additionally, similar to Pandey et al. [30] a significant positive impact of higher education on all domain scores were found in our study. Higher education and, consequently, a high monthly family income ensure that patients can access enough treatment resources, especially in countries such as Bangladesh, where most of the medical cost is spent out-of-pocket by the patients [31]. Moreover, T2DM, a chronic disease requiring lifelong adherence to medicines, continuously demands a portion of the budget reserved for healthcare costs. Patients with high monthly incomes can only achieve this.

Despite having a sound economic and educational background, complications associated with diabetes could be devastating for patients. A meta-analysis of QoL studies among diabetic patients by Jing et al. [10] identified the presence of HTN and complications as significant negative determinants of QoL in T2DM individuals. Our analysis revealed that a higher number of complications (two or more) was associated with a higher decline in QoL scores in all domains. This finding agrees with Gebremedhin et al. [25]. However, Mishra et al. [18] reported a non-significant lower QoL scores in all domains among T2DM patient compared to those with single complication. The interesting finding of our study is that after separate adjustment of microvascular (retinopathy, nephropathy, and neuropathy) and macrovascular complications (CAD, stroke, and PAD) in the multivariate linear regression model, diabetic retinopathy was found to negatively impact all domains of QoL. Secondly, PAD significantly affected all domains except physical health. On the other hand, nephropathy was associated with a significantly lower physical health and environmental domain score. CAD was found to affect psychological health more than other domains.

Previously, studies assessing QoL using WHOQOL-BREF instrument seldom explored the effect of micro- and macro-vascular complications of DM in the QoL of patients. However, other studies, using mainly the EuroQoL instrument [32] for health related QoL assessment, appear to have found different complications as significant modifiers of QoL in T2DM patients. A comparative investigation of Willige et al. [33] between EuroQoL and WHOQOL-BREF in detecting QoL have shown that both of the instruments are capable of detecting changes in physical and psychological QoL and party measures the same aspects of QoL. Hence, a discussion of the impact of diabetic complications on the QoL of patients as detected by EuroQoL or other similar instruments could be informative. Abedini et al. [34], using EQ-5D-5L instrument, found that neuropathy and nephropathy are significant associations for impaired mobility and increased pain, respectively. Meher et al. [35] used the QOLID instrument and observed that complications of T2DM involving eyes, ears, kidneys, nervous system, foot, and mind (depression), although significant in univariate analysis, become statistically nonsignificant modifier of QoL in multivariable analysis. After multivariable adjustment in their study, only foot complications and depression remained independently significant for QoL. Nevertheless, determining the independent impact of different micro- and macrovascular complications of diabetes on the QoL of patients might be difficult because they frequently occur in combinations. Redekop et al. [36], using EQ-5D-3L instrument and considering microvascular and macrovascular complications in groups, discovered that both are significant negative determinants of QoL and can have higher impacts when combined. Alshayban and Joseph [37] demonstrated similar results. However, the presence of diabetes itself could be the principal determinant of low QoL in patients rather than the presence of complications. As shown by Safita et al. [22], only eye problems remained significant among complications after adjustment for other sociodemographic variables and the presence of diabetes as a factor. In fact, proper care of diabetic patients might significantly improve their QoL. Meher et al. [35] assessed the impact of proper care in diabetic patients using a diabetic care scale and discovered a significant positive impact of good care among them. The meta-analysis of Jing et al. [35] also noted the importance of good sugar control activity in the quality of life of diabetic patients. Their study revealed physical exercise and glucose checks as significant positive modifiers of QoL. Therefore, type 2 diabetes might significantly impair quality of life without proper care and disease control activity.

Our study had certain limitations. It was conducted in a single center and was of cross-sectional nature. There were no healthy comparison groups. Only single measurements of anthropometric and biochemical profiles were used. The impact of good diabetic care on the QoL of patients could not be evaluated. However, it was one of the fewer attempts to explore the QoL of T2DM patients in its four domains using the WHOQOL-BREF instrument.

## Conclusion

This study revealed that patients with T2DM mellitus have a relatively poor QoL in physical health, psychological, social relationship and environmental domains. They have an overall bad impression about their QoL and a dissatisfaction with their health. Poor economic condition, lower education level and presence of complications are significant determinants of their QoL. Health authorities and clinicians involved in the prevention and management of diabetes should take these findings into account and incorporate necessary measures to ameliorate negative modifiers of QoL.

## Supplementary Information

**Additional file 1.** Questionnaire.

## Data Availability

The datasets used and/or analyzed during the current study available from the corresponding author on reasonable request- dr.jahid61@gmail.com.

## Abbreviations

BDT: Bangladeshi Taka
BIRDEM: Bangladesh Institute of Research and Rehabilitation in Diabetes, Endocrine and Metabolic Disorders
CAD: Coronary Artery Disease
FBS: Fasting Blood Sugar
2hABF: 2 Hour After Break Fast
HTN: Hypertension
HDL: High Density Lipoprotein
LDL: Low Density Lipoprotein
PAD: Peripheral Artery Disease
QoL: Quality of life
T2DM: Type 2 Diabetes Mellitus
TG: Triglyceride
WHOQOL-BREF: World Health Organization Quality of Life Questionnaire Brief
