# Supplementary material for: Assessment of quality of life and its determinants in type-2 diabetes patients using the WHOQOL-BREF instrument in Bangladesh
Source: BMC Endocr Disord. 2022 Jun 18;22:162. doi: 10.1186/s12902-022-01072-w (PMC9206302; doi:10.1186/s12902-022-01072-w)
Supplement: Supplementary file 1 — Additional file 1. Questionnaire. [file 12902_2022_1072_MOESM1_ESM.pdf]

Questionnaire

About you

Before getting started, we would like to know answers to some questions about you. Put a circle around the right answer or write answer in the vacant place-

What are you? Male Female

What is your date of birth? Day/month/year ----/----/----

Up to which class you have read? No education at all

Up to primary school
Up to secondary school
Up to high school
Continued after high school

What is about your marital status?

Unmarried Married but stay alone
Married Divorced
Live together like married couple Widower/widow

Does your disease exist nowadays Yes No

If you feel somewhat disorder in your
Health, then how it affects your disease ---

Current employment:

No job
Part time
Full time
Retired

Monthly income (BDT): ____

Duration of diabetes:____

Use of medication for ____________________________________Years

Current list of Medications:

____

____

Comorbidities (if any):

____

Complications (if present):

____

____

Height:____
Weight:____
BMI:____

Base-line investigations:

CBC

FBS

2-HABS

HbA1C:

Serum creatinine (mg/dl)

Urinary albumin-creatinine ratio (mcg/mg)

Lipid profile:

LDL

VLDL

TG

TC

[Please read the Instructions before fill up the rest of the questionnaire]

These questions put here are important for your life and these aims at knowing how you feel in other spheres of your life. Please answer to all questions. If you are uncertain about which may be the right answer, in that case please give that answer which to you seem the most appropriate. These answers may be the first answers as these arise sincerely in your mind.

Please while giving answers, keep in mind your life standard, hopes and pleasure and thoughts. If you are interested, then you can give answers to these questions based on results of last two weeks of your life. For example, based on last two weeks, a question may be as follows:

	Not at all	A little	Medium	Very much	In full
Do you get support from others that much support as you need?	1	2	3	4	5

After guessing how much cooperation you got from others over last two weeks, put a circle around number you choose as the best answer out of five above. Thus if you get very much support from others, then put a circle around the number 4 which is continued to the next pages:-

----

For any further queries, you can contact with:

Dr. Feroz Amin

Principle investigator

BIRDEM General Hospital, Dhaka, Bangladesh

	Not at all	A little	Medium	Very much	In full
Do you get support from others that much support as you need?	1	2	3	4	5

If in respect of the question, do you get support from others that much support as you need over last two weeks, you get no support at all, then put a circle around the number "1". Please after reading each question, evaluate your thoughts and then put a circle around each answer you think most appropriate.

	Not at all	A little	Medium	Very much	In full
How do you evaluate the importance of your life	1	2	3	4	5
	Very dissatisfied	Enough dissatisfied	Not satisfied, not dissatisfied	Enough satisfied	Very satisfied
2. How much satisfied are you over your health?	1	2	3	4	5

Following questions are set to know how you felt over last two weeks:

	Not at all	A little	Medium	Very much	In full
3. To what extent pain of your disease obstruct your activities that you are bound to do?	1	2	3	4	5
4. In maintaining everyday life, how much of medical treatment you need to take?	1	2	3	4	5
5. How much delight do you take in life?	1	2	3	4	5
6. How much you feel your life to be successful.	1	2	3	4	5
7. To what extend can you concentrate your mind on your activities?	1	2	3	4	5

	Not at all	A little	Medium	Very much	In full
8. In your everyday life, how much secure you feel yourself?	1	2	3	4	5
9. How much healthy is your physical environment?	1	2	3	4	5

The following questions have been put to you so that we may know how much over last two weeks could you do your activities with full concentration?

	Not at all	A little	Medium	Very much	In full
10. Do you enjoy sufficient delight in maintaining your everyday life?	1	2	3	4	5
11. Can you recognize your physical build (shape)?	1	2	3	4	5
12. Do you have enough money to fulfill your requirements?	1	2	3	4	5
13. In your everyday life, you need to inform yourself to others, for this how many occasions do you get?	1	2	3	4	5
14. To what extent have you chances to enjoy pastime?	1	2	3	4	5
15. To what extent do you enjoy the opportunity to move to and fro?	1	2	3	4	5

The following questions have been put to you so that we may know how much over last two weeks could you be contented with different aspects of your life?

	Very dissatisfied	Enough dissatisfied	Neither satisfied nor dissatisfied	Enough satisfied	Very satisfied
16. How much are you satisfied at your sleep?	1	2	3	4	5

	Very dissatisfied	Enough dissatisfied	Neither satisfied nor dissatisfied	Enough satisfied	Very satisfied
17. How much are you contended with your capacity of working per day?	1	2	3	4	5
18. How much are you satisfied at your working capacity?	1	2	3	4	5
19. How much are you contented with yourself?	1	2	3	4	5
20. How much are you contended with your personal enjoyment?	1	2	3	4	5
21. How much are you satisfied with your own sexual life?	1	2	3	4	5
22. How much contented are you over the assistance you get from your friends?	1	2	3	4	5
23. How much satisfied about surrounding environment, the place where you are living?	1	2	3	4	5
24. How much are you satisfied over reaching health service to your door?	1	2	3	4	5
25. How much are you	1	2	3	4	5

satisfied over your travelling and movements?					

The following questions are put to you so that we man know which things did you over the last two weeks experience and how often?

	Never	Sometimes	Often	Many many times	Always
26. How often have you experienced negative thoughts (for example sad mood, disappointment, upsetting, indifference)	1	2	3	4	5

Did anybody help you answering the questions?---------------------------

How much time did you take in filling the questions?--------------------

Any remark from you about these questions------------------------------

Thank you for your cooperation
